# Two Isomeric C16 Oxo-Fatty Acids from the Diatom *Chaetoceros karianus* Show Dual Agonist Activity towards Human Peroxisome Proliferator-Activated Receptors (PPARs) α/γ

**DOI:** 10.3390/md15060148

**Published:** 2017-05-25

**Authors:** Angel Moldes-Anaya, Thomas Sæther, Silvio Uhlig, Hilde I. Nebb, Terje Larsen, Hans C. Eilertsen, Steinar M. Paulsen

**Affiliations:** 1 Cardiovascular Research Group, Department of Medical Biology, UiT The Arctic University of Norway, 9019 Tromsø, Norway; angel.moldes-anaya@uit.no (A.M.-A.); thomas.sather@imbv.uio.no (T.S.); terje.larsen@uit.no (T.L.); 2 MabCent-SFI, UiT The Arctic University of Norway, 9019 Tromsø, Norway; 3 Department of Nutrition, Institute of Basic Medical Sciences, University of Oslo, 0313 Oslo, Norway; h.i.nebb@medisin.uio.no; 4 Section for Chemistry, Norwegian Veterinary Institute, 0106 Oslo, Norway; silvio.uhlig@vetinst.no; 5 Norwegian College of Fishery Science, UiT The Arctic University of Norway, 9019 Tromsø, Norway; hans.c.eilertsen@uit.no

**Keywords:** PPAR, dual agonist activity, metabolomics, *Chaetoceros karianus*, LC-MS^e^, NMR

## Abstract

The peroxisome proliferator-activated receptors (PPARs) function as ligand-activated transcription factors that convert signals in the form of lipids to physiological responses through the activation of metabolic target genes. Due to their key roles in lipid and carbohydrate metabolism, the PPARs are important drug targets. However, for several of the PPAR drugs currently in use, adverse side effects have been reported. In an effort to identify compounds from marine organisms that may serve as molecular scaffolds for the development of novel and safer PPAR-targeting drugs, we performed a bioassay-guided screening of organic extracts made from organisms supplied by the Norwegian Biobank of Arctic Marine Organisms (Marbank). Among several interesting hits, we identified two poorly described isomeric oxo-fatty acids from the microalgae *Chaetoceros karianus* for which we provide the first evidence that they might display dual specificity towards human PPARα and PPARγ. Principal component analysis showed that *C. karianus* stood out from other *Chaetoceros* species, both with respect to the metabolic profile and the PPAR activity. The isolation of these compounds holds the potential of uncovering a PPAR pharmacophore with tunable activity and specificity.

## 1. Introduction

The peroxisome proliferator-activated receptors (PPARs) are operating as lipid sensing receptors at the crossroads between carbohydrate and lipid homeostasis, and metabolic disorders. Functioning as ligand-activated transcription factors, they integrate signals in the form of lipids (fatty acids, phospholipids, eicosanoids, and other oxygenated fatty acids) to physiological responses through the activation of metabolic target genes [1,2]. A first step in this process is the binding of lipid ligands to the PPAR ligand-binding domain (LBD) [3,4]. In humans, the PPAR isoforms PPARα and -γ are mainly expressed in liver and adipose tissue, respectively, while PPARδ is more ubiquitously expressed [1,5]. When PPARγ is ligand-activated it induces adipocyte growth and differentiation by transcriptionally regulating target genes involved in lipogenesis and lipid storage [6,7,8]. Moreover, the activation of PPARγ maintains normal insulin sensitivity through upregulation and secretion of adipokines such as adiponectin and leptin from adipose tissue [9]. In parallel, PPARα increases lipid uptake and energy expenditure in the liver by upregulating targets involved in fatty acid transport, activation, and oxidation [10,11]. Altogether this protects the organism against high levels of circulating triacylglycerols and harmful free fatty acids [12] and ensures normal insulin function in peripheral tissues [9]. However, when adipose tissue increases past critical mass, it attracts immune cells like NK cells and macrophages, and starts to secrete pro-inflammatory cytokines like tumor necrosis factor alpha (TNF-α), interleukin-1 beta (IL-1β), and resistin, which results in suppressed insulin action and increased low density lipoprotein (LDL)-cholesterol [13,14,15,16]. Liver steatosis is another detrimental condition, fueled by PPARγ during a high-fat state [17]. This leads to reduced fatty acid oxidation and ultimately liver failure, which together with the adverse adipokine secretion exacerbates obesity.

With their key roles in lipid and sugar metabolism, the PPARs have for many years been top priority drug targets [18]. Consequently, several PPAR ligands with well-recognized biological effects are in clinical use, like the hypolipidemic fibrates, acting as PPARα activators, and the anti-diabetic thiazolidinediones (TZDs), targeting PPARγ [19,20]. However, adverse side effects have been reported, especially for the TZDs [21,22]. One way to avoid these problems has been to find or design partial [23,24] or dual [25,26] agonists which in general seems to come with fewer side effects. Thus, the aim of the present work was to identify compounds from marine organisms that regulate PPAR activity, and that may serve as molecular scaffolds for the development of novel, safer PPAR-targeting drugs.

**Figure 1:**
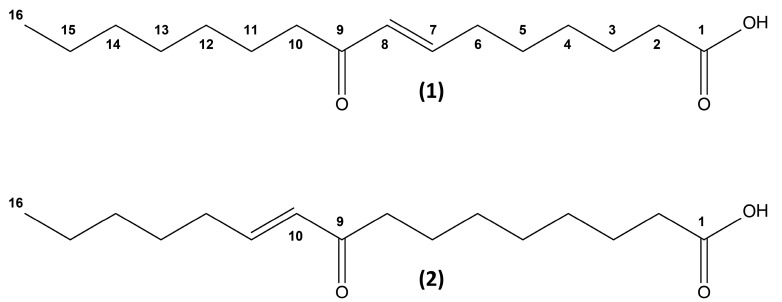
Chemical structures of the two isolated oxo-fatty acids, (7E)-9-oxohexadec-7-enoic acid (**1**) and (10E)-9-oxohexadec-10-enoic acid (**2**).

The diatom genus *Chaetoceros* contributes heavily to the overall primary production in the northern and Arctic seas [27,28]. Northern diatoms are characterized by potentially high chemical diversities and bioactivity levels [29,30]. Several species from this genus, kept in stock cultures by the Norwegian Biobank of Arctic Marine Organisms (Marbank, www.imr.no/marbank/en), were used as a library in the bioassay-guided screening for PPAR ligands reported herein. In the present study we identified two poorly described, closely related oxo-fatty acids (Figure 1) from the diatom *Chaetoceros karianus* with an apparent dual specificity towards human PPARα and PPARγ. The isolation of these isomeric compounds with dual PPAR α/γ agonist activity holds the potential of uncovering a pharmacophore with tunable activity and specificity.

## 2. Results and Discussion

The main goal of this study was to identify novel PPAR agonists or modulators from arctic marine organisms. Any such hit compound might later form the basis of a structure-activity-relationship (SAR) effort, where the aim would be to develop drugs for the treatment of diabetes and metabolic syndrome. To this end, we performed a primary screening where we transiently expressed Gal4-LDB constructs comprising the ligand binding domain (LBD) of human PPARα, -γ, and -δ fused to the yeast Gal4 DNA-binding domain in COS-1 cells, a fibroblast-like cell line derived from monkey (*Cercopithecus aethiops*) kidney. Testing of hundreds of extracts (40 mg/mL) from Marbank in the cell assay system allowed us to identify and confirm the PPAR modulatory activity from a diverse set of organisms (Table S1). The diatom species *Chaetoceros karianus* (M11031) interestingly showed a consistent activation of PPARα and -γ. Importantly, the same extracts were not able to activate human PPARδ, or human Liver X receptor (LXR) α or -β (not shown).

**Figure 2:**
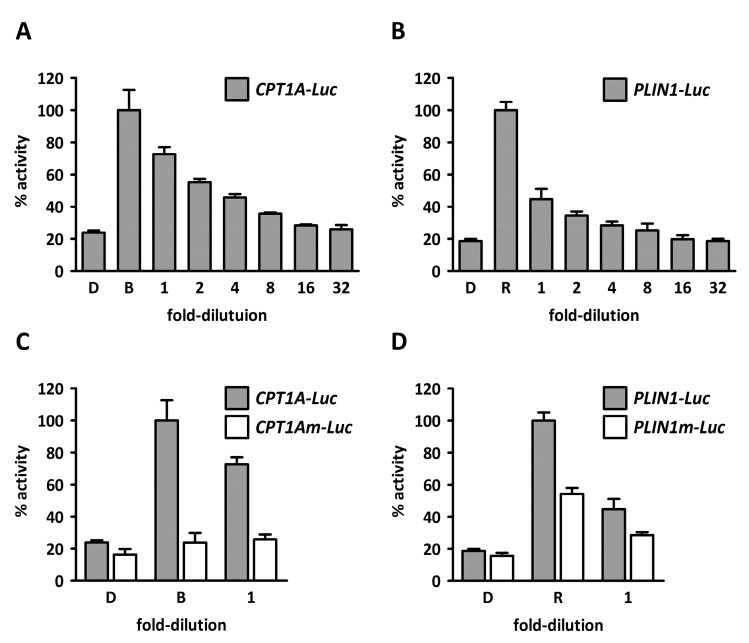
Concentration-dependent activation of natural peroxisome proliferator-activated receptor (PPAR) target gene promoters using solid phase extraction (SPE) eluates from *Chaetoceros karianus*. Monkey kidney fibroblasts, COS-1 cells, were transfected with plasmids expressing human, full-length PPARα, retinoic acid X receptor alpha (RXRα), and a *CPT1A* promoter-driven luciferase reporter (**A**) or with a *CPT1A* reporter with PPAR response elements (PPREs) mutated (**C**). Correspondingly, cells were transfected with plasmids expressing human, full-length PPARγ and RXRα and a *PLIN1* promoter-driven luciferase reporter (**B**) or with a *PLIN1* reporter with PPREs mutated (**D**). Cells were treated with a dilution series of SPE eluates (100% ACN). 1:1 dilution corresponds to ~100 μg dry weight/mL. The results represent mean relative light units (RLU) ± s.d. of two independent assays performed in duplicate. The activities of the positive controls (Rosiglitazone or Bezafibrate) were set to 100%. D: DMSO, B: 50 μM Bezafibrate, R: 1.0 μM Rosiglitazone.

To verify the findings from *C. karianus*, Flash chromatography fractions of fresh materials from the Marbank were prepared as described in the experimental section by reconstituting organic extracts in ACN/H_2_O, defatting with hexane, reconstituting in ACN/H_2_O, and eluting from a reverse-phase C18 solid phase extraction (SPE) column. The new eluates (100 mg/mL) were used to stimulate COS-1 cells, this time expressing full-length human PPARα or -γ together with retinoic acid X receptor alpha (RXRα), the natural PPAR heterodimerization partner. Gene activation was assessed on *Luciferase* reporters driven by natural promoters; human *CPTA1* for PPARα and human *PLIN1* for PPARγ. As demonstrated (Figure 2A,B), the *C. karianus* extract was able to activate both promoters. When using reporters with mutated PPAR recognition elements (PPREs), the extract’s ability to activate was significantly reduced (Figure 2C,D), corroborating PPAR-dependent agonism.

**Figure 3:**
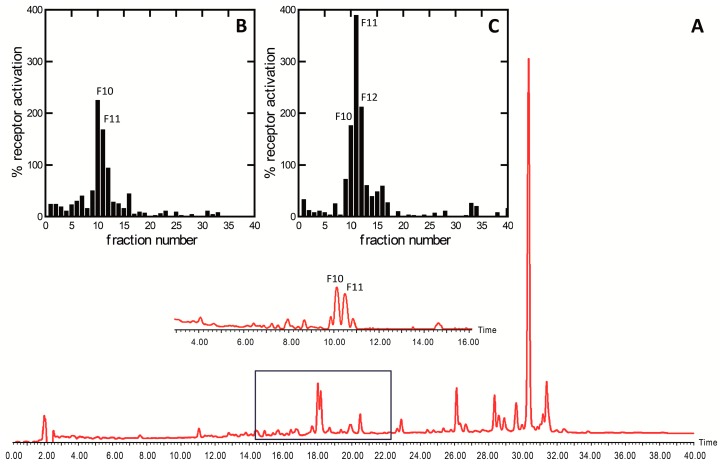
HPLC-based PPAR activity profiling of an acetonitrile/H_2_O extract of *Chaetoceros karianus.* Panel (**A**) shows a representative HPLC chromatogram (210–600 nm) of a semi-preparative separation of 1.2 g microalgal extract. The activity was initially found in the squared area in the time-based fractionation. The insert shows part of a peak-based separation for final purification. The HPLC-based activity profiling for PPARα (**B**) and PPARγ (**C**) receptor modulatory activity are shown above the chromatogram. The activities of the positive controls (Rosiglitazone or Bezafibrate) were set to 100%.

In order to track the activity found in the extract, we developed a HPLC-based activity profiling protocol (see Experimental section). The chromatogram (210–600 nm) of a semi-preparative HPLC separation (1.25 g extract) and the corresponding activity profile of the time-based fractionation (40 fractions of 60 s each) is shown in Figure 3A. The activity was found in the fractions collected between 14 and 22 min (Figure 3A). The pooled fractions of several injections were concentrated and a second semi-preparative HPLC separation based on peak signal absorption at 227 nm was applied (Figure 3, insert). The major peak of activity was found in fractions 10, 11, and 12 (Figure 3B,C). A dual, yet weakly isoform-specific, agonist activity profile was observed, where fractions 10 and 11 increased PPARα activation over the solvent controls by 224 and 167%, respectively, and fractions 10, 11, and 12 increased PPARγ activation by 175, 388, and 211%, respectively. All the remaining fractions showed minimal activity and were not considered further.

**Figure 4:**
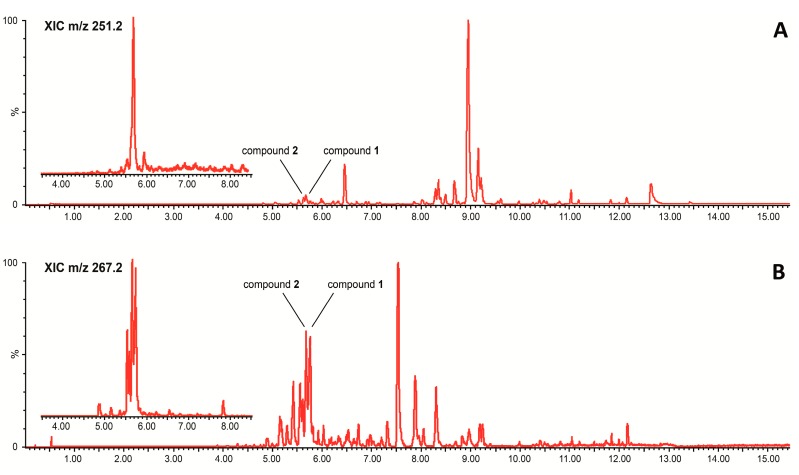
Ultrahigh performance liquid chromatography-electrospray ionization base peak chromatograms of a solid phase extraction (SPE) eluate generated from culture extracts of *Chaetoceros karianus* in (**A**) positive mode (UPLC-ESI^+^) and (**B**) negative mode (UPLC-ESI^−^). Insert: Extracted ion chromatograms of the most intense ions, *m/z* 251.2 [M + H − H_2_O]^+^ in positive mode (**A**) and *m/z* 267.2 [M − H]^−^ in negative mode (**B**).

Isolation of the two active compounds, **1** and **2**, was achieved by reverse-phase SPE and subsequent purification with semi-preparative HPLC. The compounds were tracked with the aid of ultra-performance liquid chromatography-electrospray tandem mass spectrometry (UPLC-ESIMS) and photodiode array detection (UPLC-PDA). In Figure 4A,B, typical base peak chromatograms of an SPE eluate, acquired in both positive and negative mode, are shown. The defatting step with hexane removed apolar lipids that might interfere with our screening system, while the SPE step was designed to retain polar molecules and peptides, which were not of interest in our nuclear receptor screen, as they translocate poorly to the cell nucleus and rarely have been reported to activate PPARs. As shown in Figure 4A,B, the isolated compounds ionize differently in positive and negative mode and appeared to not be the main components of the fraction mixture based on MS signal intensities. 

High-resolution mass spectrometry (HRMS) was used to identify the molecular weight and thus the molecular composition of the isolated compounds. Initial studies in positive ionization mode showed complex mass spectra for compounds **1** and **2**, indicating that it was advantageous to apply both positive and negative mode ionization protocols. Upon positive ESIMS, ions tentatively identified as [M + H]^+^ (*m/z* 269.2114 for **1**; *m/z* 269.2116 for **2**), as well as more prominent ions attributable to sodium addition ([M + Na]^+^) and dehydration ([M + H − H_2_O]^+^, [M + H − 2H_2_O]^+^), were observed (data no shown). In addition, diagnostic ions corresponding to [M + H − 46]^+^ attributable to the loss of CH_2_O_2_ indicated the presence of a carboxylic group in the two molecules. Further studies applying negative ionization mode afforded predominantly ions corresponding to [M − H]^−^ (*m/z* 267.1966 for **1** and **2**), thus confirming the identity of the [M + H]^+^ ion. This allowed us to determine the molecular formula of the two compounds, which turned out to be C_16_H_28_O_3_ for both molecules, showing that they were structural isomers with a number of ring double bond equivalents of three. 

The two isomers were subsequently determined as (7E)-9-oxohexadec-7-enoic acid (**1**) and (10E)-9-oxohexadec-10-enoic acid (**2**) from the interpretation of one- and two-dimensional NMR data as well as HRMS and HRMS/MS spectra. Furthermore, the structure determination process was assisted by database searches (Dictionary of Marine Natural Products, www.chemnetbase.com).

**Figure 5:**
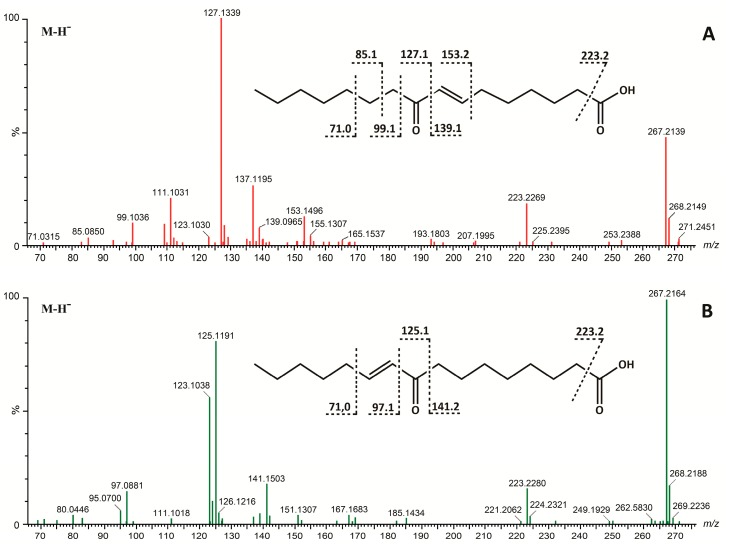
High-resolution mass spectrometry (HRMS/MS) spectra from fragmentations of [M − H]^−^ ions of (**A**) (7E)-9-oxohexadec-7-enoic acid (*m/z* 267.2139), (**B**) (10E)-9-oxohexadec-10-enoic acid (*m/z* 267.2164) and the assigned structurally informative fragmentations of these ions.

The compounds were isolated as colorless oils in quantities of 60 μg of compound **1** (approx. 95% purity) and 53 μg of compound **2** (approx. 75% purity). The UV spectra of both compounds were similar with a single absorption maximum (λ_max_) at 227 nm (**1**) and 226 nm (**2**) indicating the absence of conjugation. The HRMS/MS spectra of the two structural isomers exhibited several features that were of high diagnostic value. Most notably, fragment ions with *m/z* corresponding to [M – H − 44]^−^ upon negative ionization and [M + H − 46]^+^ upon positive ionization are commonly a result of the loss of CO_2_ and CH_2_O_2_, respectively, and indicated the presence of a carboxylic acid (Figure 5). Furthermore, the MS/MS spectra contained a series of ions typical for hydrocarbons (*m/z* 71, 85, 99), and thus gave strong evidence for compounds **1** and **2** being fatty acid derivatives. The major fragment ions, however, were observed at *m/z* 127.1339 for **1** and *m/z* 123.1038 and 125.1191 for **2** (Figure 5), indicating a structural feature in the central part of the hydrocarbon backbone that facilitates the breakage of one or more covalent bonds. 

Detailed analyses of one- and two-dimensional NMR data, including ^1^H, correlation spectroscopy (COSY), total correlation spectroscopy (TOCSY), selective TOCSY (SELTOCSY), gradient-selected heteronuclear single quantum coherence spectroscopy (g-HSQC), gradient-selected heteronuclear multiple bond correlation (g-HMBC), HSQC-TOCSY, and nuclear overhauser effect spectroscopy (NOESY) spectra determined in CD_3_CN as well as the comparison with published spectral data [31,32], afforded complete ^1^H and ^13^C assignments for compounds **1** and **2** (Table 1). The data showed the presence of a trans-coupled (^3^*J*_H-H_ = 15.9 Hz) pair of olefinic protons at 6.08/6.84 ppm in **1** and 6.08/6.85 ppm in **2**. In both isomers the lower-field olefinic proton (i.e., 6.84 ppm and 6.85 ppm) was additionally coupled (^3^*J*_H-H_ = 7.0 Hz) to a methylene proton resonance at 2.22 ppm, while the higher-field olefinic proton (i.e., 6.08 ppm) only showed weak ^4^*J*_H-H_ coupling (1.5 Hz) to the same methylene protons, but no ^3^*J*_H-H_ coupling in addition to its olefinic neighbor. At the same time, the g-HMBC spectra showed the presence of a carboxyl-carbon at 175.0 ppm (**1**) and 175.2 ppm (**2**), as well as a carbonyl-carbon at 201.4 ppm (identical shift for both compounds).

The quantity of the isolated material was too low to obtain one-dimensional direct ^13^C-NMR data, and the DEPT spectra only showed few ^13^C-resonances of low signal/noise. The carbonyl-carbon showed ^2^*J*_C-H_ and ^3^*J*_C-H_ correlations in the g-HMBC spectra to a ^3^*J*_H-H_ coupled methylene proton resonance at 2.54 ppm (identical shift for both compounds) and the lower-field olefinic proton, respectively. From these data it became clear that the carbonyl-function was interspersed between the olefinic double bond and an aliphatic chain, i.e., the compounds contained an α,β-unsaturated ketone. For further structure determination of compound **1**, the assessment of the g-HSQC, g-HMBC, and high-resolution COSY data clearly showed the presence of five methylene groups between the carboxyl-carbon and the olefinic moiety, i.e., the molecule contained a 7,8-double bond. The exact assignment of the methylene groups between the C-9 carbonyl and the terminal methyl group was ambitious, as the protons of five of the six methylene groups appeared in a narrow window between 1.29 ppm and 1.32 ppm. Using high-resolution COSY data, it was possible to identify four of the six methylene groups. Assignment of the remaining two methylene groups was only possible using the HSQC-TOCSY experiment. This is a hybrid experiment that gives through-bond correlations between a ^13^C-attached proton to all other coupled protons. The coupled protons can be seen along a line at the same ^13^C chemical shift from the carbon atom attached to the primary proton (i.e., the HSQC crosspeak). The assignment of the remaining methylene groups was then possible because individual methylene groups were well resolved in the ^13^C dimension even though they overlapped in the ^1^H dimension. NMR assignment of compound **2** started at the terminal methyl group. Long-range TOCSY data (mixing time parameter d9 = 160 ms) showed a correlation between the methyl protons and the olefinic protons, and hence these protons belonged to a common ^1^H spin system. Such a correlation was absent in a TOCSY spectrum acquired using a mixing time of 80 ms. The assignment of the methylene groups between the terminal methyl and the olefinic moiety was in this case ambitious due to the fact that the NMR sample of compound **2** contained about 25% of compound **1**. However, careful examination of the g-HMBC, g-HSQC, SELTOCSY, and COSY data starting from the terminal methyl group showed that compound **2** contained a 10,11-double bond, while the carbonyl group was at the same position as in **1**.

Mass spectrometry-aided structural elucidation of **1** and **2** was performed by interpreting HRMS and HRMS/MS analyses of the isolated compounds (Figure 5). Compound **1** showed a fragmentation pathway leading to an intense [M − 140]^−^ fragment ion apparent at *m/z* 127.1339 (Figure 5A). This ion is consistent with α-cleavage of the ketone across C8-C9, i.e., the bond joining the olefinic moiety in position C7-C8 with the carbonyl group at C9. Other minor fragments observed could be ascribed to cleavage in activated sites within the aliphatic skeleton. The fragment ion at *m/z* 139.0935 [M − 128]^−^ is likewise consistent with cleavage across the C8-C9 bond, but with the charge on the other part of the molecule (Figure 5A). The fragment ion at *m/z* 137.1195 could be explained by oxidation of the double bond at the fragment ion *m/z* 139.0935 (Figure 5A). The location of the keto group in C-9 is confirmed by the presence of the ion *m/z* 99.1036, consistent with α-cleavage of the ketone across the C9-C10 bond. Other fragment ions of interest are *m/z* 85.1, consistent with a β-cleavage at the C10-C11 bond and the fragment ion at *m/z* 153.1496 [M − 114]^−^, which could be ascribed to a cleavage of the bond adjacent to the double bond at C7-C8 (Figure 5A). Another fragment ion observed at *m/z* 223.2269 [M − 44]^−^ could be ascribed to the loss of CO_2_, which is diagnostic for carboxylic acids such as fatty acids and fatty acid derivatives. Compound **2** showed similar fragmentation pathways as compound **1** (Figure 5B), with typical α-cleavages observed in oxo-fatty acids [34]. An intense fragment ion at *m/z* 125.1191 was consistent with cleavage across C8-C9 (Figure 5B). Other important product ions were observed at *m/z* 97.0881 [M − 170]^−^, consistent with α-cleavage to the keto group across C9-C10, and the diagnostic ion at 223.2280 [M − 44]^−^, consistent with the loss of CO_2_ (Figure 5B). The prominent *m/z* 123.1038 product ion in the product ion spectrum of **2** could be the result of water loss from *m/z* 141.1503 (Figure 5B). 

As hypothesized, based on the dereplication described above, the isolated compounds turned out to be 9-oxohexadec-7-enoic acid (oxo-FA **1**) (Figure 1 and Figure 5A) and 9-oxohexadec-10-enoic acid (oxo-FA **2**) (Figure 1 and Figure 5B). After establishing the structures of the two molecules **1** and **2**, comprehensive literature searches revealed that the (E)-isomers of these molecules had recently been described from other marine organisms [31,32]. In 2005 a new C_16_ fatty acid-based oxylipin pathway was demonstrated in axenic cultures of the diatom *Thalassiosira rotula*, and compound **1** was isolated along with other novel oxo- and hydroxy unsaturated fatty acids, but no biological activity was reported [31]. Compound **2** was isolated in a later study aimed at identifying natural products with anti-inflammatory activity from the red algae *Gracilaria* verrucosa [32]. Compound **2** significantly inhibited the production of the pro-inflammatory mediators NO and IL-6 in lipopolysaccharide-activated murine macrophage cells [32].

**Figure 6:**
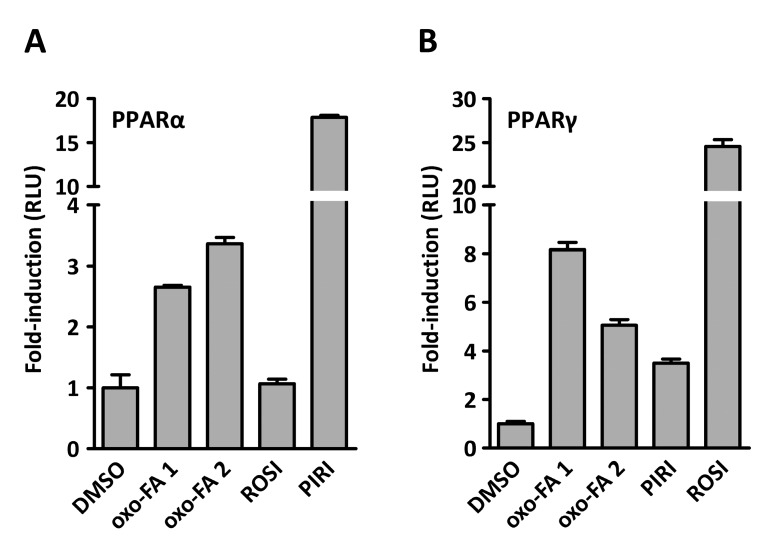
Dual PPARα/γ agonist activity with limited isoform specificity. COS-1 cells transfected with a 5 × upstream activating sequence (5×UAS)-driven luciferase reporter and (**A**) plasmids expressing yeast Gal4 DNA-binding fused to the ligand binding domain (LBD) of human PPARα (Gal4-PPARα-LBD) or (**B**) PPARγ (Gal4-PPARγ-LBD) were treated with 100 μM (7E)-9-oxohexadec-7-enoic acid (oxo-FA **1**), 100 μM (10E)-9-oxohexadec-10-enoic acid (oxo-FA **2**), 1 μM Rosiglitazone (ROSI; PPARγ agonist), or 100 μM pirinixic acid (PIRI; PPARα agonist). The results represent the mean RLU ± s.d. of two independent assays performed in quadruplicate.

Having refined the oxo-fatty acids **1** and **2** to approximately 95% and 75% purity, respectively, we wanted to reevaluate the weak PPAR isoform specificity observed during the bioassay-guided isolation. To this end we treated COS-1 cells expressing Gal4-LBD chimeras with 100 µM of **1** and **2**. As can be seen in Figure 6, the dual PPARα/γ specificity profile was retained, while the potency was somewhat reduced. Still, oxo-FA **1** seems to activate PPARγ slightly better than oxo-FA **2** (Figure 6B), while the opposite is true for PPARα (Figure 6A). The low amount of material isolated (60 µg and 53 µg of oxo-FA1 and oxo-FA2, respectively) prevented further pharmacological characterization of the two compounds. In a follow-up study, we intend to characterize the dose-response curves of these oxo-fatty acids with respect to PPAR α/γ binding and confirm the functional activation of these receptors by studying their PPAR-dependent regulation of relevant, endogenous target genes.

With their dual PPARα/γ specificity, these oxo-fatty acids might make a good start for a structure-activity-relationship (SAR) study to identify PPAR-targeting anti-diabetic drug leads. This could be achieved by comparing our oxo-fatty acids (**1** and **2**) with other cis- and trans-hexadecenoic acids that do not possess the carbonyl group in their structure. Moreover, the activity profile of the *C. karianus* oxo-fatty acids, as well as derivatives thereof, should be compared with the profile of other promising dual PPARα/γ agonists, like Saroglitazar [35]. Saroglitazar is marketed in Asia and displays significant improvement in both glycemic as well as dyslipidemic parameters with no evidence of conventional side effects (as reviewed in [25]). From a structural perspective, a recent study by Yore et al. [36] who made use of quantitative MS to identify metabolic changes in transgenic *Glut4* mice is highly interesting. Despite being obese, the studied animals showed an increased glucose tolerance. Lipidomic analysis of their adipose tissue revealed the existence of branched fatty acid esters of hydroxy fatty acids, so called FAHFAs. The most abundant form both in mice and human white and brown adipose tissue is 9-PAHSA (palmitic acid-hydroxy stearic acid), having the hydroxyl/ester group placed on the same carbon as our 9-oxo-FAs. Even though the PAHSAs signal through GPR120 to enhance insulin-stimulated glucose uptake, it could be possible that the corresponding unbranded fatty acids (with the hydroxy-/keto group on C9) might have equally beneficial effects with regard to whole body insulin sensitivity when functioning as PPAR agonists.

**Figure 7:**
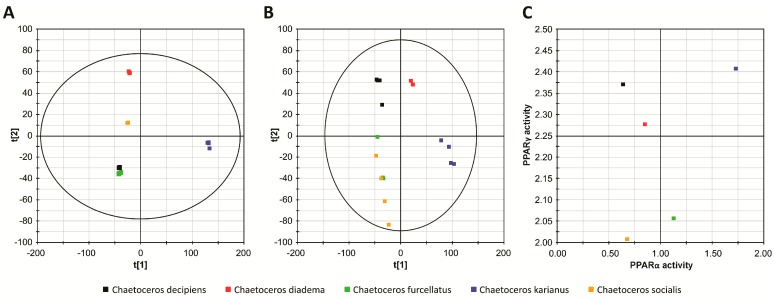
Principal component analysis (PCA) of solid phase extracts from *Chaetoceros* species shows a species-specific metabolic clustering that mirrors PPAR ligand activity from the same extracts. PCA score plots of the metabolite profile show clustering of different *Chaetoceros* species in positive ion (**A**) and negative ion (**B**) mode. Chromatograms were obtained with UPLC-MS^e^ and processed and analyzed in MarkerLynx 1.3. COS-1 cells were transfected with plasmids expressing Gal4-LBD (human PPARα and γ) and a Gal4-responsive *Luc* reporter. The cells were then treated with the same extracts as used for the PCA. The results representing the mean RLU_extract_/RLU_DMSO_ of two independent assays performed in duplicate was plotted as PPARα activity vs. PPARγ activity from the same organism (**C**).

Finally, to address whether the identified metabolite/activity profile is unique to the planktonic diatom *Chaetoceros karianus*, we employed a metabolomics approach to compare it with different, but related microalgae. Four additional strains of species belonging to the genus *Chaetoceros* were obtained from the Marbank collection. Extracts and SPE eluates were generated from *Cheatoceros decipiens* (M09048), *Chaetoceros diadema* (M09047), *Chaetoceros furcellatus* (M08023), *Chaetoceros karianus* (M11031), and *Chaetoceros socialis* (M08009) as described in the Experimental section. To assess the analogies and differences in the composition of the metabolites of the five species, a non-targeted metabolite fingerprinting of SPE eluates was conducted. ACN/H_2_O-soluble constituents from these species were analyzed by UPLC-HRESIMS as described in the Experimental Section. Results from the UPLC-HRESIMS were further processed by peak picking and alignment for further Principle Components Analyses (PCA). PCA demonstrated that striking differences exist between the metabolic profiles of the five *Chaetoceros* species (Figure 7). Interestingly, the most distinct profiles were those obtained from *C. karianus* and *C. diadema.*

When data obtained by UPLC-HRESIMS in positive ionization mode were subjected to principal component analysis (PCA) (Figure 7A), it appeared that *C. furcellatus* and *C. decipiens* are similar with regard to metabolite production. In positive ionization mode, the replicates were perfectly clustered, indicating an appropriate method repeatability. Negative ionization mode data showed similar clustering (Figure 7B), but in this case *C. furcellatus* and *C. socialis* were almost overlapping. Both in positive and negative mode, it appeared that the metabolic profiles of *C. furcellatus*, *C. decipiens*, and *C. socialis* were closely related. On the other hand *C. karianus* and *C. diadema* were less related to the other three species and to each other with regard to metabolite production. In negative ionization mode, the replicates were not as well clustered as in positive mode and showed some deviation (Figure 7B). This somehow lower reproducibility could be attributed to instrumental drift and injection problems. Interestingly, and in line with the different metabolic profiles, the different *Chaetoceros* extracts displayed different PPAR α/γ agonist activity (Figure 7C). Even though the relative position of the species was shifted, the *C. karianus* PPAR α/γ activity was found to be high and at a distance from both *C. furcellatus* and *C. socialis,* and *C. diadema* and *C. decipiens* which seemed to be more closely related, respectively. Whether this is due to differences in oxo-fatty acid levels or different levels of related or unrelated metabolites is not known and should be addressed in future studies.

*(7E)-9-Oxohexadec-7-enoic acid* (**1**) Colorless oil (60 μg). UV λ_max_ (acetonitrile/H_2_O) 227 nm. Negative HRESIMS *m/z* 267.1966; C_16_H_28_O_3_ requires *m/z* 267.1960. Positive high-resolution electrospray ionisation mass spectrometry (HRESIMS) *m/z* 269.2114; C_16_H_29_O_3_ requires *m/z* 269.2117.

*(10E)-9-Oxohexadec-10-enoic acid* (**2**) Colorless oil (53 μg). UV λ_max_ (acetonitrile/H_2_O) 226 nm. Negative HRESIMS *m/z* 267.1966; C_16_H_28_O_3_ requires *m/z* 267.1960. Positive HRESIMS *m/z* 269.2116; C_16_H_29_O_3_ requires *m/z* 269.2117.

## 3. Materials and Methods

### 3.1. Biological Material

For primary screening we used organic Flash fractions from the Marbank collection [37]. Inocula for the mass cultivation of *Chaetoceros karianus* were from our in-house culture collection. The microalga was isolated from north Norwegian spring bloom stocks in 2009. Mass cultivation was performed at 4.5 °C in a temperature and irradiation-controlled room, where the microalgae were grown in 0.3 m^3^ transparent plexiglas cylinders with external illumination. At the start of the culture period, mean scalar irradiance across the cultivation cylinder was approximately 125 μmol m^−2^·s^−1^ and at the time of harvesting it was approximately 35 μmol m^−2^·s^−1^ due to self-shadowing. The photoperiod was 14:10 (L:D). Illumination was supplied by Osram daylight fluorescent tubes.

The culture medium was Millipore filtered (0.1 μm) 33.5 ppt. seawater growth media with a concentration of inorganic nutrients equivalent to f/10 (Sigma-Aldrich, St. Louis, MO, USA) and a surplus of silicon (50 μmol). The growth rate of the cultures were monitored twice weekly by measuring the in vivo fluorescence and by microscopic inspection of the cells. The biomass concentrations at the start of the experiments were approximately 2 μg·Chl*a*·L^−1^ and by time of harvested, approximately 350 μg·Chl*a*·L^−1^. To monitor biomass development as Chl*a*, a Turner fluorometer (TD-700) was applied. The cells were harvested by sieving the culture though a 10 μm mesh size plankton net, thus letting any (small) amounts of bacteria to be flushed out. After collection, the cells were scraped off the plankton net and stored in 25 mL plastic tubes at −80 °C.

### 3.2. Bioassay-Guided Isolation Platform

#### 3.2.1. General Extract Preparation

The samples were freeze-dried, ground, and stored at −20 °C before extraction with Milli-Q water (2×, 24 h and 30 min, respectively) at 5 °C (20 mg/mL). The extracted slurries were then centrifuged and the supernatant (the H_2_O extract) was removed. The pellet was then freeze-dried and ground again.

To produce the organic extract, the pellet was extracted with MeOH/CH_2_Cl_2_ (1:1) (2× at 5 °C, 24 h and 30 min, respectively) making a 20 mg/mL organic extract. The extract was then filtered through Whatman filters (nr. 3, Ø 125 mm) and the filtrate was evaporated in vacuo and stored at −20 °C.

#### 3.2.2. Specific Extract Preparation for Bioassay-Guided Fractionation

Organic extracts (0.5–2 g) from the library prepared as described above (Section 3.2.1) were reconstituted in ACN/H_2_O (9:1) using an ultrasound bath (30 min) to make a 20 mg/mL suspension. The suspension was then filtered, recovered in a separatory funnel, and defatted with hexane (1:1, 2×). The polar organic extracts were evaporated to dryness and reconstituted in ACN/H_2_O (1:1), before being subjected to vacuum-aided solid phase extraction (SPE) on Strata C18-E cartridges (Phenomenex, Torrance, CA, USA). The eluates were recovered by a 5-fold concentration (100 mg/mL).

#### 3.2.3. Semi-Preparative High-Performance Liquid Chromatography (Semiprep HPLC-PDA)

The SPE-eluates were subjected to semi-preparative reverse-phase chromatography on a Luna C18 column (250 × 10 mm, 5 μm) (Phenomenex, Torrance, CA, USA). A 6 mL/min flow of a mobile phase consisting of 0.1% formic acid (A) and 0.1% formic acid in acetonitrile (B) was employed with gradient elution starting with 5% B, and rising to 100% B within 30 min. Then isocratic elution with 100% B was performed before switching back to the initial conditions. Time-based (60 s) fractions were collected in a Waters 2767 sample manager after detection with a Waters 2998 PDA detector (Waters, Milford, MA, USA). Fractions collected were then tested in our bioassay platform, and those showing significant activity were pooled and subjected to a second round of semiprep HPLC-PDA. In this second step, we applied a gradient elution program starting with 70% A (10 mM ammonium formate and 0.1% formic acid) and 30% B (10 mM ammonium formate and 0.1% formic acid in acetonitrile), rising to 100% B within 30 min. Then isocratic elution with 100% B was performed before switching back to the initial conditions. Compounds **1** and **2** were collected in separate fractions by following the PDA signal trace.

#### 3.2.4. Plasmids

The pSG5-Gal4-hPPARα-LBD, pSG5-Gal4-hPPARδ-LBD, and pSG5-Gal4-hPPARγ-LBD encoding Gal4 DNA-binding domain (DBD; aa 1–147) fused in frame with the SV40 nuclear localization signal 1, and the ligand binding domain (LBD) of human PPARα (aa 168–468), PPARδ (aa 140–441), and PPARγ (aa 205–505), respectively, were received as generous gifts from Dr. Krister Bamberg (AstraZeneca, Mölndal, Sweden). The same was true for the pGL3-5×UAS-SV40 luciferase reporter consisting of five Upstream Activation Sequences (UAS; 25 bp phasing) and a SV40 early promoter in front of the Firefly *Luciferase* gene. The plasmid encoding full-length human PPARγ, pcDNA3.1-hPPARg2-FLAG, was made by PCR amplification of human *PPARG* cDNA, using custom-made primers (harboring kozak and FLAG-tag), and subcloning it into pcDNA3.1(+) (Life Technologies, Carlsbad, CA, USA) between *Nhe*I and *Not*I. The corresponding PPARα plasmid, pcDNA3.1-hPPARa-FLAG, was made by PCR amplification of human *PPARA cDNA* and subcloning it into pcDNA3.1-hPPARg2-FLAG between *Nhe*I and *BamH*I, exchanging *PPARG with PPARA*, and keeping the FLAG tag. The plasmid encoding full-length human RXRα, pcDNA3-hRXRα, has been described earlier [38]. The human *PLIN1*-driven reporters, pGL3-hPLIN1-3′del and pGL3-hPLIN1-3′del-PPREmut, have been described before [7]. The human *CPT1A*-driven reporters, pGL3-hCPT1AInt and pGL3-hCPT1AInt-PPREmut, were received as a gift from Prof. Diego Haro Bautista and have been described previously [39]. The vector pRL-CMV (Promega, Madison, WI, USA), constitutively expressing Renilla *Luciferase*, was used as a control of transfection efficiency. All cloned plasmids have been sequenced. Cloning primer sequences are available upon request.

#### 3.2.5. Cell Culture, Transfection, and Luciferase Assays

COS-1 cells (ATCC^®^ CRL-1650) were maintained in DMEM (Life Technologies, Carlsbad, CA, USA) containing gentamicin (10 μg/mL) or penicillin/streptomycin (50 U/mL; 50 μg/mL) and 10% fetal bovine serum (F7524; Sigma), at 37 °C in a humidified atmosphere of 5% CO_2_ in air. Cell confluence never exceeded 80% before subculturing or transfection. For the screening campaign the COS-1 cells were transiently transfected with 1.7 µg of the Gal4-PPAR-LBD expression plasmids and 8.5 µg of the 5×UAS-SV40 luciferase reporter per 1 × 10^7^ cells, using the Neon Transfection System (Life Technologies, Carlsbad, CA, USA). The cells were then plated at 2 × 10^4^ cells/well in white 96-well plates (F96, Nalge Nunc Int., Rochester, NY, USA) and allowed to attach (5 h) before the different fractions were added at two concentrations (final DMSO concentration of 0.35% or 0.70%). Corresponding volumes of DMSO was used as the negative control. As positive controls, the following synthetic agonist were used; PPARα: Bezafibrate (BM-15075; B7273, Sigma-Aldrich, St. Louis, MO, USA) or Pirinixic acid (WY-14643; C7081, Sigma-Aldrich, St. Louis, MO, USA), PPARδ: GSK-516 (GW-501516; 43732, Sigma-Aldrich, St. Louis, MO, USA), and PPARγ: Rosiglitazone (BRL-49653; Cayman Chemical, Ann Arbor, MI, USA). After 18 h the cells were washed with PBS, before D-luciferin potassium salt dissolved in 30 mM HEPES (0.12 mg/mL), supplemented with 1 mM MgSO_4_ and 0.5 mM adenosine triphosphate was added directly to the wells (50 µL/well). Following 10 min of incubation at room temperature, the emitted light signal was measured on an Envision reader (PerkinElmer, Turku, Finland).

For the verification of hits, dose-response assays, and specificity assays, COS-1 cells were seeded at 7 × 10^4^ cells/well in 24-well plates. After 24 h, the cells were transfected with 0.1 µg of the Gal4-DBD-PPAR-LBD expression plasmids, 0.2 µg of the 5×UAS-SV40 luciferase reporter, and 0.05 µg of the *Renilla Luciferase*-coding internal control using Lipofectamin 2000 (Life Technologies, Carlsbad, CA, USA). After 5 h, the fractions or semi-pure compounds in DMSO (final conc. 0.7%) were added to the culture and the incubation continued overnight. After 18 h, the cells were washed in PBS and lysed in Passive Lysis Buffer (Promega, Madison, WI, USA) and the Dual-Luciferase^®^ ReporterAssay System (Promega, Madison, WI, USA) was run on a Synergy 2 plate reader (BioTek, Winooski, VT, USA) following the manufacturer’s protocol. The Firefly Luciferase readings were normalized to the Renilla Luciferase numbers and data from at least two independent transfection experiments, run in duplicate, are presented.

### 3.3. Structure Elucidation Platform

#### 3.3.1. Ultra-Performance Liquid Chromatography Coupled to Data-Independent Mass Spectrometry (UPLC-MS^e^)

High resolution mass spectra (UPLC-QToFMS) were recorded in positive and negative mode, making use of both electrospray (ESI) and atmospheric pressure chemical ionization (APCI) interfaces on a Xevo G2 QToF mass spectrometer (Waters, Milford, MA, USA). The following settings were applied in both ESI negative and ESI positive MS: Capillary voltage 0.5 kV, source and desolvation temperatures of 130 °C and 400 °C, respectively, cone and desolvation gas at 20 L/h and 800 L/h, respectively. The instrument was run in MS^e^ resolution mode with a scan time of 0.1 s, centroid mode. Low energy (no collision energy) and high energy (ramp collision energy from 30 to 50 V) spectra were acquired in the mass range from *m/z* 100–1200. The instrument was regularly calibrated with sodium formate with leucine enkephaline applied as a lock mass.

Chromatographic analysis was performed using an Aquity 50 × 2.1 mm (i.d.) 1.7 μm UPLC BEH C18 column (Waters, Milford, MA, USA) with a mobile phase consisting of 0.1% formic acid (A) and 0.1% formic acid in acetonitrile (B). Different linear gradient programs were applied for scouting and compound-directed chromatography. The longer scouting program applied in the initial studies on complex mixtures was as follows: 90% A was kept for the first 2 min, then elution from 10 to 100% B over 25 min with a 10 min hold at 100% B before the eluent was switched back to the initial conditions. The shorter program was as follows: 98% A kept constant for 2 min, then elution from 2 to 100% B over 3 min and hold at 100% B for 2 min before switching back to the initial conditions. A mobile phase flow rate of 0.6 mL/min was used, and 2–10 μL samples were injected.

#### 3.3.2. Ultra-Performance Liquid Chromatography Coupled to Photodiode Array Detection (UPLC-PDA)

The chromatographic features applied in the UPLC-PDA analysis were similar to those described above (Section 3.3.1), including column type, mobile phase, and elution properties. The UV spectra were recorded with an Acquity PDA detector (Waters, Milford, MA, USA) between 210 and 600 nm. The wavelength resolution was 1.2 nm. 

#### 3.3.3. NMR Spectroscopy

NMR spectra of compounds **1** and **2** were obtained from solutions (500 µL) in acetonitrile-*d*_3_ (CD_3_CN, 99.96 atom % D; Sigma-Aldrich, St. Louis, MO, USA) using 5 mm o.d. Wilmad tubes (Sigma-Aldrich, St. Louis, MO, USA). The spectra were acquired on an Avance AVII 600 MHz NMR spectrometer (Bruker BioSpin GmbH, Rheinstetten, Germany) equipped with a 5 mm CP-TCI triple resonance inverse cryoprobe with a Z-gradient coil. NMR assignments were obtained from the examination of ^1^H, DEPT135, 1-D SELTOCSY, COSY, TOCSY, g-HSQC, g-HMBC, HSQC-TOCSY, and NOESY NMR spectra. The data were processed using Bruker TOPSPIN (version 2.1 pl4 or version 3) software. Chemical shifts, determined at 25 °C, were reported relative to internal C*H*D2CN (1.96 ppm) and *C*D3CN (118.26 ppm).

### 3.4. Metabolomics

Diluted SPE-eluates (1 μg/mL) of the species *C. decipiens*, *C. diadema, C. furcellatus, C. karianus*, and *C. socialis* were analyzed following the protocol described above (Section 3.3.1) in UPLC-MS^e^ in both ESI positive and negative mode. The chromatograms obtained from the UPLC-MS^e^ analyses were processed by the MarkerLynx 1.3 application manager (Waters, Milford, MA, USA) for mass signal extraction and alignment. MarkerLynx makes use of ApexTrack for peak detection and integration, listing the detected peaks as their *m/z* values and retention times along with their associated intensities. When the data are collected, they are automatically delivered to the Extended Statistics software integrated in MarkerLynx and multivariate statistics are applied for unsupervised principal components analyses (PCA). The parameters applied in the MarkerLynx application manager were as follows: XIC windows, 0.02 Da; marker intensity, 1200; mass windows, 0.02 Da; retention window, 0.1; the Apex Track peak parameters were automatically calculated by MarkerLynx. The isotopes were not included in the peak detection process.

## 4. Conclusions

We have identified two isomeric oxo-fatty acids from *Chaetoceros karianus* with apparent dual PPARα/γ agonist activity. These oxo-fatty acids also show a limited isoform-specificity. (7E)-9-oxohexadec-7-enoic acid mainly activates PPARγ, while (10E)-9-oxohexadec-10-enoic acid primarily activates PPARα. Principal component analyses indicate that *C. karianus* differs from other *Chaetoceros* species, both with respect to the metabolic profile and the PPAR activity. This finding holds the potential of defining a PPAR pharmacophore with tunable activity and specificity.

## Figures and Tables

**Table 1:** ^1^H and ^13^C NMR data of compounds **1** and **2** (600/150 MHz in CD_3_CN, δ in ppm, *J* in Hz).

Position	1 ^a^		2 ^a^	
	^13^C	^1^H	^13^C	^1^H
1	175.0, t	-	175.2, t	-
2	34.2, t	2.27, t, (7.4)	34.3, t	2.27, t, (7.5)
3	25.6, t	1.57, m	25.6, t	1.57, m
4	29.5, t	1.34, m	29.8, t	1.31, m, overlapped
5	28.7, t	1.48, m	29.8, t	1.31, m, overlapped
6	33.0, t	2.22, ddt, (7.5, 7.0, 1.5)	29.8, t	1.31, m, overlapped
7	148.2, d	6.84, dt, (15.9, 7.0)	25.1, t	1.55, m
8	131.1, d	131.1, dt, (15.9, 1.5)	40.4, t	2.54, t, (7.4)
9	201.4, s	-	201.4, s	-
10	40.4, t	2.54, t, (7.4)	131.2, d	6.08, dt, (15.9, 1.5)
11	25.1, t	1.55, m	148.1, d	6.85, dt, (15.9, 7.0)
12	29.8, t	1.30, m	33.0, t	2.22, ddt. (7.5, 7.0, 1.5)
13	30.4, t	1.29, m	28.6, t	1.48, tt, (7.5, 7.5)
14	32.5, t	1.30, m	32.2, t	1.33, m
15	23.3, t	1.32, m	23.3, t	1.34, m
16	14.4, q	0.90, t, (7.0)	14.4, q	0.91, t, (7.0)

^a^ chemical shifts, determined at 25 °C, are relative to internal C*H*D_2_CN (1.96 ppm) and *C*D_3_CN (118.26 ppm) [33].

## Supplementary Materials

The following are available online at www.mdpi.com/1660-3397/15/6/148/s1, Table S1: PPAR agonist activity found in the Marbank collection.
