# Supplementary material for: Two Isomeric C16 Oxo-Fatty Acids from the Diatom Chaetoceros karianus Show Dual Agonist Activity towards Human Peroxisome Proliferator-Activated Receptors (PPARs) α/γ
Source: Mar Drugs. 2017 May 25;15(6):148. doi: 10.3390/md15060148 (PMC5484098; doi:10.3390/md15060148)
Supplement: Supplementary file 1 [file marinedrugs-15-00148-s001.pdf]

marine drugs

Supplementary Information

Two isomeric C_{16} oxo-fatty acids from the diatom *Chaetoceros karianus* show dual agonist activity towards human peroxisome proliferator-activated receptors (PPARs) α/γ

Angel Moldes-Anaya[1,2], Thomas Sæther[1,3], Silvio Uhlig[4], Hilde I. Nebb[3], Terje Larsen[1], Hans C. Eilertsen[5], Steinar M. Paulsen[2,*]

[1] Cardiovascular Research Group, Department of Medical Biology, UiT The Arctic University of Norway, Tromsø, Norway.
[2] MabCent-SFI, UiT The Arctic University of Norway, Tromsø, Norway.
[3] Department of Nutrition, Institute of Basic Medical Sciences, University of Oslo, Oslo, Norway.
[4] Section for Chemistry and Toxicology, Norwegian Veterinary Institute, Oslo, Norway.
[5] Department of Arctic and Marine Biology, UiT The Arctic University of Norway, Tromsø, Norway

* Correspondence: Steinar M. Paulsen; E-mail: steinar.paulsen@uit.no; Tel.: +47-776-44504

Academic Editor: name
Received: date; Accepted: date; Published: date

List of tables in supporting information:

Table S1. PPAR agonist activity found in the Marbank collection of invertebrates and diatoms

Table S1. PPAR agonist activity found in the Marbank collection of invertebrates and diatoms

Marbank ID	Organism	PPARα	PPARδ	PPARγ
M10004-0-L03	*Actiniaria sp.*	nt	+	+
M11002-0-L03	*Alaria esculenta*	-	-	-
M11006-0-L03	*Alcyonium digitatum*	nt	nt	-
M10010-0-L03	*Astarte crenata*	nt	++	++
M10066-0-L03	*Atlantopandalus propinqvus*	++	-	++
M11016-0-L03	*Attheya longicornis*	++	++	++
M10051-1-L03	*Attheya longicornis* (0 min UV light exp)	++	-	++
M10062-1-L03	*Attheya longicornis* (110 min UV light exp)	+++	-	-
M10056-1-L03	*Attheya longicornis* (120 min UV light exp)	++	-	-
M10058-1-L03	*Attheya longicornis* (165 min UV light exp)	+	-	++
M10059-1-L03	*Attheya longicornis* (180 min UV light exp)	-	-	+
M10057-1-L03	*Attheya longicornis* (60 min UV light exp)	+	+	+++
M10055-1-L03	*Attheya longicornis*, MI #2, (0 min UV light exp)	-	+	+
M10067-0-L03	*Aurelia aurita*	++	-	-
M11017-0-L03	*Bacteriosira bathyomphala*	+++	-	+++
M11031-0-L03	*Chaetoceros karianus*	+++	-	+++
M10013-1-L03	*Chimaera monstrosa*	+	-	-
M11003-0-L03	*Cnidaria indet*	nt	nt	-
M10001-0-L03	*Craniella polyura*	nt	-	+
M11001-0-L03	*Cyanea capillata*	++	-	++
M11030-0-L03	*Cylindrotheca closterium*	++	++	+++
M10027-1-L03	*Echinus-a esculentus*, MI #1	nt	-	-
M10027-2-L03	*Echinus-a esculentus*, MI #2	nt	-	+
M10027-3-L03	*Echinus-a esculentus*, MI #3	nt	-	+
M10065-1-L03	*Filograna implexa*	++	-	++
M10030-0-L03	*Fucus distichus*	-	-	-
M10031-0-L03	*Fucus serratus*	-	-	-
M10007-0-L03	*Gastropoda indet*, eggs	nt	+	-
M11040-0-L03	*Geodia barretti*	nt	nt	++
M11018-0-L03	*Halichondria panicea*	-	+	+
M10038-0-L03	*Haliclona (rhizoniera) rosea*	nt	-	+
M10005-0-L03	*Hormathia nodosa*, MI #1	nt	+	++
M10006-0-L03	*Hormathia nodosa*, MI #2	nt	-	++
M10002-0-L03	*Hymenaster pellucidus*	nt	+	-
M10034-0-L03	*Kukenthalia borealis*	nt	-	+
M10033-0-L03	*Laminaria hyperborea*	nt	-	++

M11024-0-L03	*Molgula retortiformis*	nt	nt	+
M10063-0-L03	*Munida sarsi*	-	+	+
M10014-0-L03	*Mycale (mycale) lingua*	-	-	+
M11015-0-L03	*Myxilla sp.*	nt	nt	+
M11029-0-L03	*Odontella aurita (hl,*	++	-	-
M11007-0-L03	*Ophioscolex glacialis*	nt	nt	+
M11026-0-L03	*Pelvetia canaliculata*	nt	nt	++
M11008-0-L03	*Phakellia ventilabrum*	nt	nt	+
M10003-0-L03	*Polymastia boletiformis*, MI #1	nt	-	-
M11036-0-L03	*Polymastia boletiformis*, MI #2	nt	nt	++
M10017-0-L03	*Polymastia sp.*	+	-	++
M10044-0-L03	*Polysiphonia lanosa*	+	-	-
M11019-0-L03	*Pontaster tenuispinus*	-	-	-
M10029-0-L03	*Porifera indet.* A	+	-	++
M10039-0-L03	*Porifera indet.* B	nt	-	++
M11009-0-L03	*Porifera indet.* C	nt	nt	+
M11010-0-L03	*Porifera indet.* D	nt	nt	+
M11011-0-L03	*Porifera indet.* E	nt	nt	-
M11012-0-L03	*Porifera indet.* F	nt	nt	-
M11013-0-L03	*Porifera indet.* G	nt	nt	-
M11014-0-L03	*Porifera indet.* H	nt	nt	-
M11027-0-L03	*Porifera indet.* I	nt	nt	-
M11035-0-L03	*Porifera indet.* J	nt	nt	++
M11037-0-L03	*Porifera indet.* K	nt	nt	++
M11038-0-L03	*Porifera indet.* L	nt	nt	+
M11039-0-L03	*Porifera indet.* M	nt	nt	++
M11041-0-L03	*Porifera indet.* M	nt	nt	+
M11042-0-L03	*Porifera indet.* N	nt	nt	-
M11043-0-L03	*Porifera indet.* O	nt	nt	++
M11045-0-L03	*Porifera indet.* P	nt	nt	++
M11005-0-L03	*Porifera indet.* Q	nt	nt	-
M11033-0-L03	*Porosira glacialis*, MI #1 (UV)	-	++	-
M11034-0-L03	*Porosira glacialis*, MI #2 (UV)	-	-	+
M10064-0-L03	*Pseudarchaster parelii*	++	-	-
M10032-0-L03	*Psolus phantapus*	-	+	-
M10035-1-L03	*Saccharina latissima*, MI #1	nt	-	++
M10035-2-L03	*Saccharina latissima*, MI #2	nt	-	-
M10035-3-L03	*Saccharina latissima*, MI #3	nt	-	+++
M10008-0-L03	*Scaphander lignarius*	nt	-	+

M10028-1-L03	*Solaster endeca*, MI #1	nt	-	-
M10028-2-L03	*Solaster endeca*, MI #2	+++	++	-
M10028-3-L03	*Solaster endeca*, MI #3	-	-	++
M11028-0-L03	*Solaster endeca*, MI #4	-	-	-
M11028-2-L03	*Solaster endeca*, MI #5	-	-	-
M11028-3-L03	*Solaster endeca*, MI #6	++	++	-
M10016-0-L03	*Styela rustica*	+	-	+
M10036-0-L03	*Tethya norvegica*	nt	-	-
M11032-0-L03	*Thalassiosira antarctica var. borealis*	-	-	-

+ : activity screening, ++ : activity re-testing, +++ : activity in HPLC fractions, - : no activity, nt : not tested, MI: Material Identity (sampled at different locations, depths, time of year etc.)

© 2017 by the authors. Submitted for possible open access publication under the terms and conditions of the Creative Commons Attribution (CC BY) license (http://creativecommons.org/licenses/by/4.0/).
